# Development of Kampo（traditional Japanese medicine）e-learning program: evaluation of the flipped classroom for medical students

**DOI:** 10.1080/10872981.2021.1938504

**Published:** 2021-06-16

**Authors:** Aki Ito, Kenji Watanabe, Yoshitaka Fukuzawa, Kazuo Mitani, Shinichi Fujimoto, Takahide Matsuda, Kiyoshi Sugiyama, Kiyoshi Kitamura, Nobutaro Ban

**Affiliations:** a Human Resources Development and Training Department, Kanagawa Institute of Industrial Science and Technology, Kanagawa, Japan; b Center for Kampo Medicine, Keio University School of Medicine, Tokyo, Japan; c Aichi Medical Preemptive and Integrative Medicine Center (AMPIMEC, Aichi Medical University School of Medicine, Aichi, Japan; d Nara Medical University, Nara, Japan; e Department of Internal Medicine, Yamatokashihara Hospital, Nara, Japan; f Division of General Internal Medicine, St. Marianna University School of Medicine, Kanagawa, Japan; g Department of Clinical Pharmacokinetics, Hoshi University, Tokyo, Japan; h Institute of Community Medicine, Japan Association for Development of Community Medicine, Tokyo, Japan; i Medical Education Center, Aichi Medical University School of Medicine, Aichi, Japan

**Keywords:** Kampo, traditional medicine, flipped classroom, e-learning, medical school

## Abstract

In May 2019, the World Health Assembly, in an unprecedented move, endorsed the inclusion of traditional medicine in the International Classification of Diseases, 11th Revision. In Japan, traditional medicine (known as Kampo) is regulated by the government and prescribed by over 90% of physicians along with modern medicine under the national health insurance system. Although Kampo education must be included in Japan’s core medical curricula, there are significant challenges to implementation. In the educational context, the flipped classroom teaching method has received considerable attention in recent years. This study developed a Kampo e-learning program and verified the effectiveness of a flipped classroom using Kampo e-learning. The Kampo e-learning Committee determined three courses and assigned an administrator for each. The administrators appointed lecturers who developed Kampo e-learning lessons. Physicians, pharmacists, medical students, and pharmacy students were asked to participate in the e-learning program, and their comments and suggestions were collected after program completion. The flipped classroom was evaluated by implementing Kampo e-learning in the Kampo session with fourth-year students at Keio University School of Medicine in Japan. Seven courses were created, including four based on volunteer suggestions. The ‘Systematic Kampo Curricula’ featured 88 lessons developed by 54 Kampo specialists. Out of 118 fourth-year medical students who participated in the flipped classroom, 113 registered for the Kampo e-learning program, 100 attended the session, and 88 answered the post-session questionnaire. Among the students who answered the questionnaire, 86.4% were satisfied with the flipped classroom, 79.5% replied that the program made them understand Kampo and 80.7% stated that it should be adopted. The flipped classroom using Kampo e-learning program was shown to be attractive in one medical school. Further expanded study is necessary in the near future to reveal the usefulness of the flipped classroom of Kampo learning.

## Introduction

There is an increasing global demand for traditional medicine as people seek alternatives to modern medicine in managing their health. In response to this demand, the classification for traditional medicine has been included for the first time in the eleventh version of the International Statistical Classification of Diseases and Related Health Problems (ICD-11), which was endorsed at the 72nd World Health Assembly in May 2019.

Kampo is the Japanese modification of traditional medicine classified in the ICD-11, and is used widely in the Japanese healthcare system. In Japan, only one type of medical license is issued allowing physicians to implement both modern biomedicine and traditional medicine under the National Health Insurance System. Unlike in other countries, such as the USA and European nations where most herbal preparations are regulated as dietary supplements, Kampo formulas are regulated in Japan as pharmaceutical grade prescription drugs [1].

Under these circumstances, over 90% of Japanese physicians prescribe Kampo in their daily practice. Moreover, approximately 70% of physicians prescribe Kampo for cancer patients to alleviate the adverse effects of chemotherapy or radiation therapy and improve quality of life [2–6]. However, since the national examination for physicians only covers modern biomedicine, medical school curricula focus mostly on this aspect of medicine. For this reason, most physicians prescribe Kampo formulas based on their knowledge of modern biomedicine rather than Kampo theory. In fact, we have previously clarified that 90% of physicians have prescribed Kampo, while less than 30% have learned Kampo theory [7]. For example, Kakkonto is a traditional formula prescribed by many physicians to treat the common cold. However, Kampo experts prepare individualized formulas based on Kampo theory, knowing that this approach will result in a better outcome for the patient. Therefore, Kampo knowledge is essential when preparing Kampo formulas [1,8].

In 2001, the Kampo curricula became a mandatory component of medical education when the Ministry of Education, Culture, Sports, Science, and Technology-Japan (MEXT) developed the ‘Model Core Curricula for Medical Education (2016 revision) in Japan,’ which included the requisite minimum content that every medical student must learn. The objective of Kampo education in the Model Core Curricula is to ‘outline the characteristics of Kampo medicine, and indication and pharmacological effects of major Kampo formulas’ [9].

A diagnostic system at the center of Kampo theory allows students to outline the characteristics of Kampo. In particular, it is essential that students are able to identify the unique Kampo ‘pattern diagnosis.’ Pattern diagnosis refers to ‘the complete clinical presentation of the patient at a given moment in time including all findings. Findings may include symptomology or patient constitution, among other things’ [10–12]. There are three big challenges to learning Kampo theory in the medical education system: a shortage of Kampo education sessions, a shortage of Kampo expert educators, and a lack of standardized educational materials and methods [13].

To overcome these problems, we have established a web-based Kampo e-learning program to be implemented with a flipped classroom model. Since the introduction of the flipped classroom in 2000 [14], its effects have been reported in various fields worldwide. The ‘flipped classroom’ is the commonly used term for a pedagogical model in which didactic lecture and homework elements are reversed [15,16].

Since 2012, numerous publications have focused on the flipped classroom method. As many educational institutions currently use the flipped classroom, numerous studies have been conducted to examine the effects of this model. The percentage of students who attended the biochemistry dissertation course, ‘The class without lectures,’ offered by Stanford University School of Medicine, increased dramatically from about 30% to 80%, even though class attendance was optional [17]. In an introductory lecture on circuit analysis at San Jose State University, the pass rate increased from 59% to 91% after the flipped classroom was adopted [18]. Other studies have also improved student performance after introduction of the flipped classroom [18–21]. Previous research has also shown that after participating in the flipped class, students reported that they had ‘enjoyed’ the class, were ‘actively participating,’ had ‘autonomy in learning,’ and experienced an increase in their ‘motivation to learn’ [22,23].

This study presents our Kampo e-learning program, which was developed with the support of a Grant-in-Aid from MEXT. We conducted a feasibility study of the first flipped classroom implementing Kampo e-learning program and have evaluated the participants’ feedback.

## Methods

### Development of Kampo e-learning program

The Kampo e-learning Committee was established with seven physicians and one pharmacist to cover both medical and pharmaceutical education requirements. The Committee created three Kampo e-learning courses: ‘Kampo examination’ ‘Systematic Kampo Curricula,’ and ‘Reference Materials.’ Kampo e-learning courses were delivered by the Learning Management System (LMS) platform developed by SATT, Inc, Tokyo, Japan, to facilitate access to course content on smartphones, tablets, and PCs.

The Kampo e-learning Committee selected the e-learning courses as follows: (i) The ‘Kampo examination’ course consisted of a video lecture about the Kampo examination and was presented by a physician. (ii) The ‘Systematic Kampo Curricula’ course comprised five units: history, Kampo theory, pharmacognosy, Kampo formulas, and Kampo clinical application. The Committee selected one administrator for each unit who determined the lesson topics. The administrators selected 54 lecturers from all over Japan, including physicians, dentists, and pharmacists specializing in Kampo. These lecturers then created a total of 88 lessons which were divided by the units. Four of the clinical application lessons covered the field of dentistry. The lectures consisted of PowerPoint slideshows with audio narration. The length of a single lecture was five minutes for medical school students and 15 minutes for continuing medical education for physicians. The lecturers also developed review questions for each lecture. The lessons for medical school students had two questions per lecture (total: 176); the lessons for continuing medical education for physicians had five questions per lecture (total: 440). (iii) The ‘Reference Materials’ course included descriptions of 180 crude drugs and 224 Kampo formulas that appeared in all lectures. The crude drug materials consisted of two types of photos: pieces of crude drugs for decoction and whole crude drugs. The crude drug materials also included information published in the Japanese Pharmacopoeia: title name, English name, Latin name, commonly used name, and family name of the medicinal plant. The Kampo formula materials provided the names of crude drugs used in Kampo formulas along with photos. We also linked these materials to the crude drugs and Kampo formulas that were featured in the online lectures.

During the development of Kampo e-learning program, we asked physicians, pharmacists, medical students, and pharmacy students to participate in the e-learning courses, and collected their comments and suggestions after program completion. The Committee subsequently made improvements to the e-learning content based upon participant feedback.

### Evaluation of the flipped classroom using the Kampo e-learning program in medical school

The flipped classroom session was conducted in the Kampo class for fourth-year students (n = 118) at Keio University School of Medicine. The Kampo class comprised eight sessions; ‘Role and basic concept of Kampo medicine,’ ‘Kampo examination 1,’ ‘Kampo examination 2,’ ‘Kampo treatment for digestive and respiratory diseases,’ ‘Kampo medicine treatment for gynecological diseases,’ ‘Acupuncture and moxibustion,’ ‘Kampo medicine treatment for cancer patients and elderly patients’ and ‘Caution of Kampo formulas and crude drugs.’ There were 90 minutes designated per session, for a total of 12 hours. The flipped classroom was held on the subject of digestive disorders from the fourth session on 12 January 2016. Students were notified by the Student Affairs Division that they would register for Kampo e-learning on January 5 and must participate in the study’s designated e-learning content by January 12. It was also announced that the Kampo e-learning system could be accessed on smartphones, tablets, and PCs.

Students were required to complete five designated contents: the (i) ‘Kampo examination’ course and four lessons (‘Chronic gastritis,’ ‘Loss of appetite,’ ‘Diarrhea,’ and ‘Constipation’) from the (ii) ‘Systematic Kampo Curricula’ in preparation for the flipped classroom session. It took about 20 minutes to complete the ‘Kampo examination’ course, and it took about five minutes to complete a lesson on the ‘Systematic Kampo Curricula.’ Students registered in the e-learning system were able to take not only the designated content but also all the courses shown in (Table 1Table 2) whenever they wanted to access them.

**Table 1.** Evaluation comments

Kampo diagnostic examinations It was easier to imagine how to perform the examinations, dispense medicine and give patient compliance instructions by seeing them done on video.
Systematic Kampo curricula It was easier to understand it through e-learning than by attending the lesson in a traditional classroom. It was easier to understand Kampo terms when described verbally, because Kampo text contains many difficult kanji characters. It was easy to learn because we could watch the screen and understand the lecture delivered through voice. It was easy to concentrate on, understand, and remember the lecture content because the volume of content to be handled per session had been narrowed down. It was convenient and easy to access the lesson using a mobile phone. It felt like there were little review questions asked. It would be more enjoyable if there were questions with accompanying photos, for example, ‘What is this crude drug?’ It might be helpful to test us for the final exams and to test us on past questions of national exams. It is better to include more practical questions and to confirm our knowledge through a question and answer session. It is better to provide slides and transcripts of the lecture.
Reference materials It was helpful to link the lecture to the commentary page with photos.

**Table 2.** Kampo e-learning course based on feedback

(i) Kampo diagnostic examinations
(ii) Systematic Kampo curriculum: 88 lessons History (2) Kampo theory (22) Phamacognosy (10) Kampo formula (20) Kampo clinical application (34)
(iii) Reference materials Kampo formulas (224) Crude drugs (180)
(iv) Kampo quizzes What is this crude drug? (Looking at a photo) What is this Kampo formula? (Looking at the composition of crude drugs) What is the Kampo formula in this text? (Looking at the original text)
(v) National examination - National Examination for Pharmacists
(vi) Botanical gardens Makino Botanical Garden Tokyo Metropolitan Botanical Garden Tsukuba Botanical Garden
(vii) Archival contents Lectures by the late Keisetsu Otsuka

In the flipped classroom session, the teacher distributed a printout of three case summaries (Appendix) to students. The students participated in small group discussions (6–10 students) about the three cases and the teacher asked questions for 15 minutes. Subsequently, for each question, the group representatives presented their thoughts on the rationality of their answers. After the presentation, the teacher and students discussed the clinical reasoning of the cases for 40 minutes. After this discussion, the teacher gave a supplementary lecture during the remaining time.

Students responded to the web questionnaire-based evaluation of the flipped classroom from February 1 to 4 March 2016. A final exam of Kampo class was conducted by a written exam on 3 March 2016.

The facilitating university determined that it was not necessary for the ethical review committee to review the questionnaire.

The collected answers of the questionnaire and the score of the final exam were analyzed using JMP® (SAS Institute Inc., Cary, NC, USA).

## Results

### Revision of Kampo e-learning program

Feedback on the courses was collected from volunteers consisting of nine physicians, three pharmacists, nine medical students, and 75 pharmaceutical students (Table 1). Based on these comments, scripts of the lectures were prepared for all lessons in the ‘Systematic Kampo Curricula’ course, and four new courses were created (Table 2). In the new Kampo quiz course, 10 questions were randomly chosen from many questions to create each of three types of quizzes: ‘What is this crude drug?’ ‘What is this Kampo formula?’ ‘What is the original textbook of this Kampo formula?’ A commentary lecture on Kampo medicine, crude drugs, and natural compounds was prepared as a national examination course for pharmacists. In addition, courses were created covering medicinal plant botanical gardens and archival content.

### Evaluation of the flipped classroom

**Figure 1.**
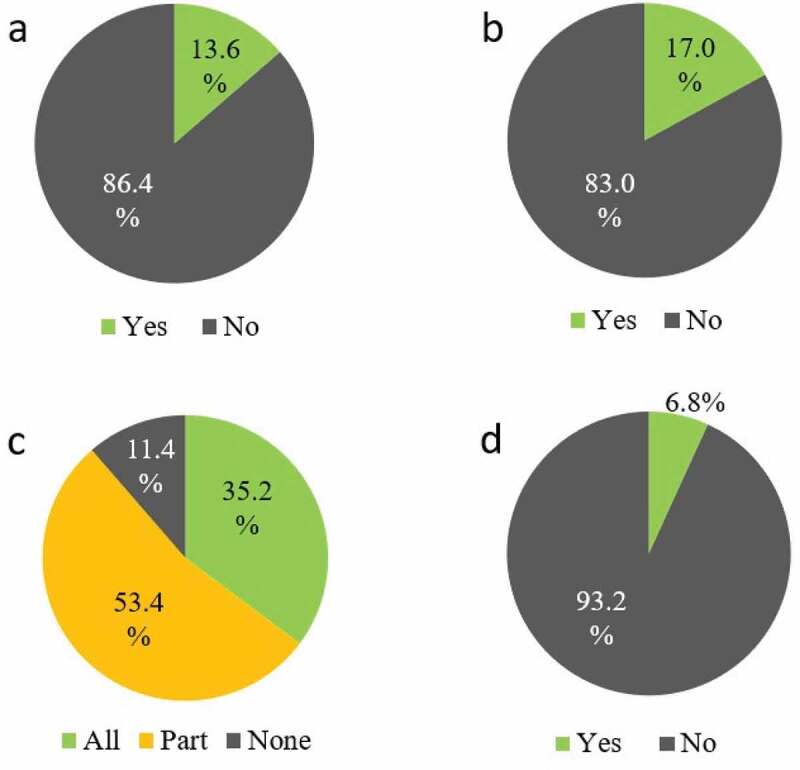
Experience of flipped classroom and completion rate of e-learning contents (n = 88)

In total, 113 (95.8%) students enrolled in the e-learning system, 100 (84.7%) attended the sessions, and 88 (74.6%) responded to the post-session web questionnaire (Table 3). Seventy-six students (86.4%) had never heard of the flipped classroom model, and 73 (83.0%) reported they participated in a flipped classroom for the first time. In total, 31 (35.2%) students completed the five designated contents: the ‘Kampo examination’ course and four lessons (‘Chronic gastritis,’ ‘Loss of appetite,’ ‘Diarrhea,’ and ‘Constipation’) from the ‘Systematic Kampo Curricula.’ Forty-seven (53.4%) students completed one to four lessons, and 10 (11.4%) did not complete any. Approximately 60％ of students who did not complete the designated 5 contents mentioned a lack of time as the reason. Half of these students said they needed to prepare for computer-based testing (CBT) which was scheduled soon after the flipped classroom session. Passing the CBT is mandatory for fourth-year students to be able to advance to the fifth year. On the contrary, six students completed some other contents in addition to the five designated contents (Figure 1).

**Table 3.** Student background (N = 118)

Number of registered students on the e-learning system	113 (95.8%)
Number of attendees in the session	100 (84.7%)
Number of respondents to the web questionnaire	88 (74.6%)

(a) Have you ever heard of ‘flipped classroom’?(b) Have you ever participated in a flipped classroom session?(c) Did you complete the designated 5 contents?(d) Did you complete some of the other contents?

**Figure 2.**
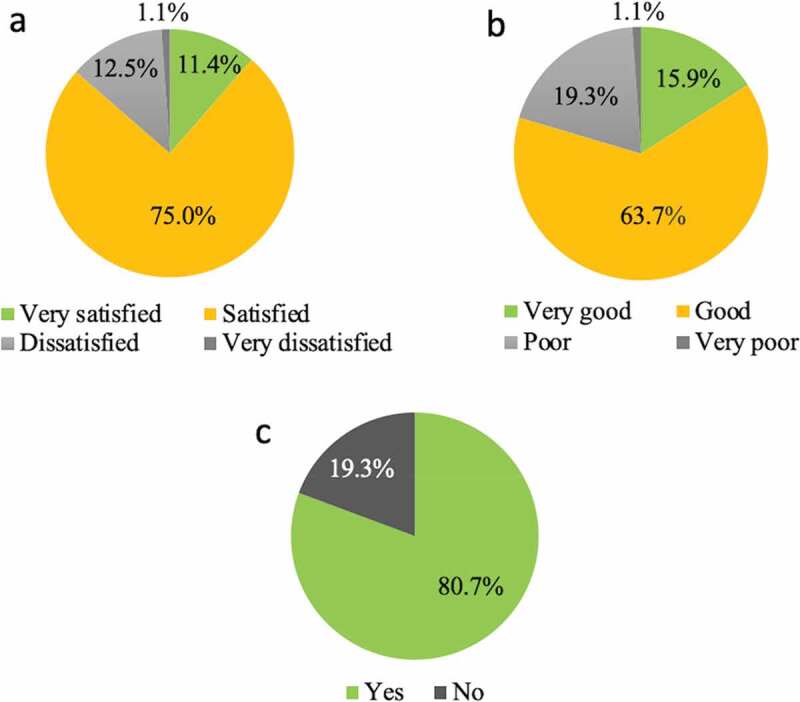
Students’ answers about FC (n = 88)

Regarding student satisfaction with the course, 10 (11.4%) were ‘very satisfied,’ 66 (75.0%) were ‘satisfied,’ 11 (12.5%) were ‘dissatisfied,’ and one (1.1%) was ‘very dissatisfied.’ Regarding comprehension rate, 14 (15.9%) stated that it was ‘very good,’ 56 (63.6%) said it was ‘good,’ 17 (19.3%) said it was ‘poor,’ and one student (1.1%) said it was ‘very poor.’ Seventy-one (80.7%) students believed that ‘the flipped classroom should be adopted,’ and 17 (19.3%) thought that ‘the flipped classroom should not be adopted’ (Figure 2). In the ‘Comprehension’ item, a significant difference was seen between the ‘Completing all five online contents’ group and the ‘Not completing all five online contents’ group (Table 4).

**Table 4.** Comparing the item ‘completing designated five contents’ against other items

Items	Completing	Agree	Disagree	P-value
Satisfaction	All None	29 8	2 2	0.2093
Comprehension	All None	28 6	3 4	0.0267*
Adoption	All None	25 8	6 2	0.9643

(a) Please rate your satisfaction with the session.(b) Please rate your comprehension level.(c) Do you think the flipped classroom must be adopted?

## Discussion

### Development of Kampo e-learning program

For the first time, we have successfully developed an e-learning program for mandatory Kampo education in all Japanese medical schools to solve three major problems: a shortage of sessions, a shortage of educators, and a lack of standardized educational materials and methods [13].

Kampo examinations of patients are carried out differently than modern medicine examinations, and employ a unique method called the ‘Four Examinations (Looking, Hearing and Smelling, Questionnaires and Palpation)’. Understanding the Kampo examination is difficult by textbook study only. It has been reported that learning via video can make the content easier for students to understand [24,25]. The ‘Kampo examination’ course, which is a video-based lecture, was evaluated by students as allowing for easy comprehension through the use of actual images.

Lectures in the ‘Systematic Kampo Curricula’ course consisted of Power Point slideshows with the lecturer’s audio narration and a script. According to the Learning Pyramid by National Training Laboratory of Bethel, Maine, auditory learning increases understanding, and the retention of knowledge is better than visual learning with textbook. Therefore, all lectures in the ‘Systematic Kampo Medicine Curricula’ course were accompanied by audio. Students requested the script for the lectures, reporting that ‘It’s helpful to understand the lectures.’ Since some Kampo terms are not familiar to medical students, it is often the case that the pronunciation is not understood if only the letters are seen, and vice versa. Based on these opinions, we decided to add audio scripts to all lectures. Therefore, we thought that combining text and audio would be helpful for students learning Kampo. Student evaluations of the transcripts described them as ‘helpful to understand the lectures’, and evaluations of the audio narration stated that ‘Study with auditory sensation is more effective than textbooks.’ It is generally said that student concentration lasts less than 15 minute [26]. Therefore, the lecture length of the ‘Systematic Kampo Curricula’ course was appropriate as students were able to maintain their concentration and easily comprehend the material. As a result, the lectures were evaluated as being effective for student retention of course content. Further, by providing review questions for each lecture, the course enabled students to verify their mastery of the subject matter while proceeding with the content.

Kampo formulas consist of crude drugs, creating the risk of drug overlap when several Kampo formulas are prescribed together, which may result in overdose [27]. For example, since licorice is used in 70% of Kampo formulas, an overdose of licorice can easily occur when multiple formulas are prescribed at the same time, causing pseudoaldosteronism. Therefore, it is important for students to learn the crude drugs used in Kampo formulas [27]. To reinforce comprehension of these drugs, we linked crude drug information to the Kampo formulas taught in the lectures. Student evaluations were high for the linked information.

The Kampo e-learning program was developed in Japanese, and in consideration of the inclusion of Kampo medicine in the ICD-11, an English version is under preparation.

### Evaluation of the flipped classroom

In this study, the overall high student evaluation for the flipped classroom was similar to findings of previous studies in several areas [28–31]. Students showed high levels of satisfaction (86.4%) and comprehension (79.6%). Seventy-one (80.7%) students stated that ‘the flipped classroom should be adopted.’ Students discussed cases and questions based on the clinical reasoning approach in the flipped classroom session. We did not collect the data for responses to questions because we emphasized the thinking process of resolving case studies. However, it was possible to hold active discussions since students had acquired knowledge of Kampo theory through e-learning. Therefore, it was considered that students highly evaluated the flipped classroom in terms of satisfaction, comprehension, and adopting.

There were three students in the ‘satisfied’ and ‘good level of comprehension’ categories who had completed all five designated contents, but who nevertheless answered that ‘the flipped classrooms should not be adopted.’ All three stated that the timing of the Kampo session was too close to the CBT. However, it was not possible to reschedule the program. Therefore, when we use a flipped classroom we have to be cognizant of what else is currently competing for student time. We infer that if the Kampo session was not scheduled just before the CBT, student satisfaction and comprehension rates would be higher. Also, more than 80% of the students answered that the flipped classroom should be adopted in the future. This means that the flipped classroom was accepted by most students. Thirty-one students (35.2%) took all five designated contents, meaning that a majority of the students studied only some of the courses. The students who could not attend any courses and those who did not complete all contents stated a shortage of time as the reason. Nearly half of these students again complained that the timing was close to the CBT. On the other hand, there were a few students who had learned more than the designated contents even in the situation before CBT (Figure 1(d)). It was meaningful to provide an environment in which students could voluntarily learn content other than what was designated.

Since the completion rate was related to the level of comprehension, it is necessary to increase the completion rate in order to increase the level of comprehension.

All medical students are required to pass the CBT to advance to the fifth year, but Kampo content is not included in the CBT. Therefore, it seems that some students could not afford to do the required preparation before the session. However, it takes approximately 40 minutes per week, or less than 10 minutes per day, to complete the ‘Kampo examination’ course (about 20 minutes) and four lessons (about 5 minutes each) from the ‘Systematic Kampo Curricula.’ The time should not have placed a great burden on the students. Nonetheless, the students had not been notified about the time required for the designated contents during the study, and so it appears that the students felt burdened. For this reason, we suggest that advance notification of the required preparation time would increase students’ completion rate of e-learning contents. On the other hand, even though the preparation time is at least 40 minutes, it seems that some students spent more time. In the future, we should understand their schedules outside class and consider their overall study schedule. In addition, we will verify the appropriate preparation time and content for students before the flipped classroom, such as previous reports [22,32–34].

Previous studies have reported an increase in attendance rate, pass rate and examination scores with the implementation of a flipped classroom [17,18,35,36]. This study implemented a flipped classroom in one of the eight Kampo sessions, and the attendance rate was 84.7%. Since attending the Kampo sessions was a degree credit requirement, the attendance rate was high for the other seven sessions as well. The pass rate is 90% every year, and there was no change in the rate the year the study was conducted. Since this study was not designed to analyze the scores of the final exam as evaluation items, it was difficult to compare the scores of the traditional classroom in the previous year with the flipped classroom. However, in the final exam (100-point scale), the score of the ‘Completing all five online contents’ group (75.9 points) was higher than the ‘Not completing all five online contents’ group (71.1 points), although there was no significant difference. In the future, we will compare the final examination score of the traditional classroom with that of the flipped classroom. The following comments were received from students: ‘We should continue the flipped classroom in the future,’ ‘the flipped classroom was fun.’ However, the students were not asked about ‘active participation,’ ‘autonomy of learning,’ and ‘motivation to learn’; these outcomes will be investigated in the future research.

Based on the study findings, we concluded that our Kampo e-learning program implemented in the flipped classroom was useful for the Kampo session. A typical problem in the flipped classroom model is the development of teaching materials prior to the class [37]. However, teacher burden was eliminated in this study because the information and communication technology (ICT) teaching materials for systematic Kampo learning had already been completed in the MEXT project. Also, since the e-learning lectures were created by 54 Japanese Kampo experts, content quality was not an issue. Furthermore, this pilot study received high evaluations from students regarding satisfaction and comprehension.

The limitation of this study is that it was implemented at only one medical school. We are planning to increase the number of participating medical schools in Japan for further evaluation. In addition, although there are only subjective data of students, objective data such as the results of the final exam [36,38] will be designed as evaluation items to compare the traditional classroom and the flipped classroom in the next study.

A flipped classroom is a type of blended learning of online and face-to-face education. The Kampo e-learning program that we developed can be used as online education, and we aim to verify its effects in the future.

## Conclusion

To improve the standard of Kampo education in medical schools, as well as in continuing education programs for physicians, this study successfully developed the Kampo e-learning program, which included the ‘Kampo examination’ with a video lecture, the ‘Systematic Kampo Curricula’ with 88 lessons created by Kampo experts, and ‘Reference Materials’ linked to online lectures. The study results indicated that students found the flipped classroom using Kampo e-learning program to be highly beneficial. The flipped classroom using Kampo e-learning program was shown to be attractive in one medical school. The main factor is that the Kampo e-learning Committee, an organization of medical education professionals and Kampo medicine professionals, has developed the standardized program. Further expanded study is necessary in the near future to reveal the usefulness of the flipped classroom of Kampo learning.

## Prior poster presentation

This work was presented as poster presentations at the 2017 International Congress on Complementary Medicine Research and the 2017 International Society for Japanese Kampo Medicine.

## Appendix Case summaries

**Table:**

Case 1.	Patient: Female, 74 years old When the patient was 68 years old, she underwent surgery for ascending colon cancer. After the surgery, she was constipated and felt cold in her lower abdominal region. She was hospitalized three times and recovered by fasting. After taking purgative medication, she suffered from lower abdominal pain and could not move, and was sensitive to cold. Height: 148 cm; Weight: 38 kg; Pulse: 68/min and normal; Blood pressure 132/68; Signs of weak abdominal muscles Q: What is her pattern? a. Deficiency b. Excess c. Medium Q: What is her pattern? a. Cold b. Heat c. Moderate d. Both cold and heat Q: What should you prescribe? a. 麻子仁丸 (Mashiningan) b. 大柴胡湯 (Daisaikoto) c. 大建中湯 (Daikenchuto) d. 防風通聖散 (Bofutsushosan) e. 大黄牡丹皮湯 (Daiobotampito)
Case 2.	Patient: Female, 68 years old She has had weak digestion since she was a child. For the past two months, she has been very worried, and then she began to have stomach aches, which began before meals and disappeared after eating. She underwent a gastro camera, had redness and vascular permeability in the pylorus area, and was diagnosed with chronic gastritis. Anorexia was noted during these two months, and her weight dropped by 2 kg. She was prescribed a proton pump inhibitor, and this treatment relieved her stomach ache, but she still had no appetite. Height: 154 cm; Weight: 46 kg; Pulse: 84/min and normal; Blood pressure 124/70; Signs of weak abdominal muscles. No signs of 胸脇苦満(hypochondrium resistance and discomfort). Q: What should you prescribe? a. 四逆散 (Shigyakusan) b. 安中散 (Anchusan) c. 平胃散 (Heiisan) d. 大建中湯 (Daikenchuto) e. 黄連解毒湯 (Orengedokuto)
Case 3.	Patient: Male infant, three years old The fever began last night, and vomiting and diarrhea appeared this morning. Vomiting occurred suddenly, and it occurs after the patient drinks water. Recently, several cases of rotavirus infection have appeared in nursery schools. The body temperature is 39.1°C. Q: What should you prescribe? a. 葛根湯 (Kakkonto) b. 真武湯 (Shimbuto) c. 五苓散 (Goreisan) d. 半夏瀉心湯 (Hangeshashinto) e. 大黄牡丹皮湯 (Daiobotanpito)
